# Social Identity, Core Self-Evaluation, School Adaptation, and Mental Health Problems in Migrant Children in China: A Chain Mediation Model

**DOI:** 10.3390/ijerph192416645

**Published:** 2022-12-11

**Authors:** Ye Chen, Xinxin Yu, Aini Azeqa Ma’rof, Zeinab Zaremohzzabieh, Haslinda Abdullah, Hanina Halimatusaadiah Hamsan, Lyuci Zhang

**Affiliations:** 1 Faculty of Human Ecology, Universiti Putra Malaysia, Serdang 43400, Malaysia; 2 Department of Education, Guangxi Normal University, Guilin 541004, China; 3 Institute for Social Science Studies, Universiti Putra Malaysia, Serdang 43400, Malaysia; 4 Faculty of Educational Studies, Universiti Putra Malaysia, Serdang 43400, Malaysia

**Keywords:** core self-evaluation, mental health, migrant children, school adaptation, social identity

## Abstract

(1) Background: The present study investigated the relationships between social identity, core self-evaluation, school adaptation, and mental health problems in migrant children, and the mechanism underlying these relationships; (2) Methods: The participants were migrant middle school students in China. Data analysis was conducted using SPSS version 26. A survey comprising the social identity scale, core self-evaluation scale, school adaptation scale, and mental health scale MMHI-60 was deployed; (3) Results: Findings indicated a significant and negative association between social identity and mental health problems, and such an association was sequentially mediated by core self-evaluation and school adaptation. Furthermore, core self-evaluation and school adaptation played a chain mediation role between social identity and migrant children’s mental health problems; (4) Conclusions: It is crucial to improve social identity, core self-evaluation, and school adaptation to reduce mental health problems among this population. Therefore, the research results provide a new direction for promoting the development of mental health education for migrant workers and their children in China.

## 1. Introduction

The year 1979 marked the birth of modern China with exponential growth and changes in its economy and society, facilitated by reforms and the opening up of borders [1]. This created a never-before-seen social trend of the rapid growth of the migrant population. When 2016 came to a close, official reports showed that there were a staggering 245 million internal migrants across China, specifically in cities. Indeed, 75% of these migrants moved from rural regions to urban centers, and they were profiled in the category of low education attainment and skills [2]. The sheer scale of this migration movement has created a new generation of demographics that continues to attract attention. Specifically, in the urban centers, the 2010 national census reported that there were as many as 35.81 million migrant children [3,4]. Historically, the growth of this population saw an increment of 27.80% between 2000 and 2005 and 41.37% in the period 2005 to 2010 [5]. Compared with their parents who were born and bred in the countryside, most migrant children have been dwelling in cities for a good number of years. In a 2013 study, researchers revealed that on average, migrant children’s years of residence in their city destination was 3.74 years, with one-third of them reported at least six years of residence [6].

Various studies have shown that migration and the process itself significantly impact the mental state of children and adolescents [7,8,9]. In particular, this underscores the inherent mental health vulnerability of China’s rural-to-urban migrant children; they experience depression, loneliness, social anxiety, and low self-esteem [10]. With a dataset of more than 4600 migrant children and 5000 urban children, a meta-analysis revealed that migrant children reported significantly more serious mental health problems compared with their peers who are urban natives [11]. The growing body of literature on mental health models continues to explore the use of a multi-dimensional model to predict a wide range of well-being outcomes [12,13]. It has been demonstrated that the dimension of social identity has a direct impact on mental illness symptoms such as depression [14,15,16], paranoid ideation [17], anxiety [18], well-being, and post-traumatic stress [16].

Social identity is an important dimension of psychological functioning [19]. When the sense of group belonging is internalized, it profoundly influences an individual’s thoughts, feelings, and actions. Therefore, social identity provides critical access to social support that enhances overall health. Students with social identities are reported to be more grounded and anchored with a sense of ‘existential security’—a mental state that Durkheim [20] explained as capable of giving more strength, fulfillment, and resiliency when challenges and vulnerability beset individuals. Based on further theoretical studies on social identity and its connection to health, researchers extended its conceptualization into health contexts [21]. For our current study, Jetten’s [22] social cure model of health serves exceptional importance because it places social identification as central to health and well-being outcomes. This model stipulates that when people are socially connected to a group, the group identity integrates with their self-identity. The group, through this process of social identification, provides a psychological resource that has a positive impact on health, both directly and indirectly [23]. The centrality of social identities at the heart of mental health and well-being [24] therefore allows its psychological resource conceptualization in migrant children, therein serves to explain how increasing a sense of belonging and self-worth can fortify them against distress.

Two other predictors of healthy psychological functioning are school adaptation and core self-evaluation [25]. School adaptation refers to the ability to adapt to the school, to feel comfortable, to commit, and to accept its social context [26]. This suggests the interfusion of cognition, attitude, and behavior. Researchers have highlighted that a healthy mental state potentially helps school-goers deal or cope with school demands, manage resources, and adopt a positive attitude toward school [27]. Kor [28] revealed how closely connected mental health, well-being, and middle-schoolers adaptability to school were. In addition, Steinmayr [29] established school adaptation as a predictor of mental health. The second predictor, core self-evaluation, refers to the evaluation of one’s ability and self-worth, and its close connection to depression has been reported [30]. Furthermore, core self-evaluation plays two mediating roles—the first between stressful life events and depression, and the second between shyness and depression [31,32,33]. Past studies have also shown that core self-evaluations negatively correlate with mental health symptoms [34]. Accordingly, when school-going children display high core self-evaluation, they not only exude confidence, but they believe that with their strong sense of control, they can overcome most things in life [35], hence they experience reduced stress and mental health problems due to lack of control.

However, our current extant knowledge found the absence of studies that examine the influencing role of social identity in children’s mental health, specifically in China’s migrant school-going children and adolescents. Responding with a thorough literature review, we pursue focus on investigating both core evaluation and school adaptation factors that could contribute to the relationship between social identity and mental health of this cohort. Therefore, we examine the influencing role of social identity on migrant children’s mental health issues impacted by core self-evaluation and school adaptation; specifically, we scrutinize the indirect influence mechanism of social identity on the study cohort’s mental health issues. Essentially, this study attempts to address research gaps in this area; mental health literature and research in China have largely neglected the examination of rural-to-urban migrant children [36,37]. Past research on this study cohort had primarily focused on educational outcomes, namely education rights, dropout rates, and educational consequences [38,39]. There are limited studies that are concerned about this cohort’s mental health [40,41,42]. With these findings, we offer recommendations on health and social support required to develop this cohort’s mental health progressively. Thus, the present investigation aims to test the following hypotheses:

**Hypothesis** **1** **(H1).** *There is a negative relationship between social identity and migrant children’s mental health problems.*

**Hypothesis** **2** **(H2).** *School adaptation mediates the negative relationship between social identity and migrant children’s mental health problems.*

**Hypothesis** **3** **(H3).** *Core self-evaluation mediates the negative relationship between social identity and migrant children’s mental health problems.*

**Hypothesis** **4** **(H4).** *School adaptation and core self-evaluation create a chain mediating effect on the impact of social identity on migrant children’s mental health problems.*

## 2. Materials and Methods

### 2.1. Study Design

A cross-sectional study was conducted to investigate the chain mediating effects of school adaptation and core self-evaluation on the relationship between social identity and mental health problems in migrant children. This study design was paired with a survey in the synchronous investigation and description of the variables in focus.

### 2.2. Participants and Procedures

A total of 900 migrant middle-schoolers aged between 12 and 17 years participated in this study, and 852 (94.6%) usable questionnaires were returned. Participants were recruited via a multistage cluster random sampling technique. This technique aimed at ensuring equal opportunity of selection was employed [43]. We first segmented 14 cities in the Guangxi Zhuang Autonomous Region in China, wherein a large population of floating residents resides. China’s floating residents comprise residents who move away from their registered hukou location [44]. Four cities from Guangxi were randomly selected, including Nanning, Guilin, Beihai, and Qinzhou. From each city, four secondary schools were chosen. First, second, and third grades are found in every secondary school. From each grade, one class is chosen at random. All classes selected from each school were eligible to be sampled until the number of students in each school was satisfied to 900. Data were first checked for normality using the histogram and normal Q-Q plots of probability, then the Kolmogorov–Smirnov (K-S) test was used to determine the application of parametric testing. After the outliers were eliminated, 827 of the 852 students were included in the research.

Guided by their classroom teachers, the students spent 40 min of class time answering the questionnaire, and the questionnaires were collected uniformly after class. Biases of many kinds that influence the subjects of every research may keep them from answering the questions honestly (e.g., social desirability bias) [45]. It is possible to handle these biases in a number of ways, but one of them is to reassure responders that there are no right or incorrect responses. As a result, they will not have to stress over coming up with a socially acceptable response since they will know that any response is absolutely fine.

The researchers obtained prior informed consent from both the participants and their guardians before this survey was conducted. In this study, mean substitution was used in this investigation to fill in the gaps left by missing data. In mean substitution, the mean of the indicator’s valid values is used to replace the missing value. When the missing values for each indicator are less than 5%, mean substitution can be used, according to Hair [46].

Results of descriptive statistics and analysis of the study population are shown in Table 1. Table 1 shows the number proportion of migrant middle-schoolers by gender, grade level, and time of migration, as well as the status of social identity and mental health problems. There were 465 boys (56.2%) and 362 girls (43.8%) in the sample, including 264 (31.9%) in the first year of junior middle school, 283 (34.2%) in the second year, and 280 (30.9%) in the third year. Some 88 (10.6%) migrated for half a year to 1 year, 207 (25.0%) for 1 to 3 years, 159 (19.2%) for 3 to 5 years, and 373 (45.1%) for more than 5 years.

The evaluation of demographic variables in consideration was conducted using an independent sample *t*-test and one-way ANOVA, e.g., [47,48]. One-way ANOVA was employed to assess the dissimilarities between the social identity scale, the school adaptation scale, the core self-evaluation scale, and the MMHI-60 scale among different demographic characteristics (i.e., migration time and grade). The findings showed that the social identity of the female cohort was significantly higher than that of their male counterparts (*p* = 0.000). There are significant differences in the level of social identity among migration junior high school students with different living times (*p* = 0.049). The least significant difference (LSD) was found that the social identity level of migration junior high school students with a migration time of half a year to one year was lower than that of migration junior high school students with a migration time of 3 to 5 years and more than 5 years.

### 2.3. Measurements

Social identity was measured using Ward and Kennedy’s [49] Acculturation Index (AI) which was revised into the Chinese version. Originally, the scale provides two measurable dimensions, namely identification with the culture of origin and identification with the host culture. A total of 21 cognitive and behavioral items are offered, including “in-group and out-group perception”. With a 7-point scale, the participants rate 1 (the least similar) to 7 (extremely similar) when comparing living in the culture of origin and the culture of the host country. The Cronbach’s alpha coefficient was 0.773.

The school adaptation scale measured how well middle-schoolers adapted to school for five years in the aftermath of an earthquake in China’s Yushu Tibetan Autonomous Prefecture in 2010. The Cronbach’s alpha was 0.78 [50]. The measuring quality of this scale was validated via a preliminary survey conducted during the pre-investigation of Tibetan adolescents. The test-retest reliability was 0.82 while the content validity of the scale was 0.89.

The core self-evaluation scale is a mono-dimensional scale that measures core self-evaluation, which Zenger [51] compiled into 10 items. To modify and adapt this scale to be used in the Chinese cultural context, Du [52] developed a translated and revised version based on relevant theories and research. This scale provides a five-level score that ranges between 1 (complete disagreement) and 5 (complete agreement). The total score is as low as 10 and as high as 50 points; a higher score indicates a higher level of core self-evaluation. The Cronbach’s alpha coefficient of this scale was 0.83, where the split-half reliability was 0.84, and the test-retest reliability of the 3-week interval was 0.82.

Mental health problems were measured using the validated scale called the Mental Health Inventory of Middle School Students (MMHI-60). This 60-item scale was a compilation by Wang [53] for participants to self-report their mental health problems. This scale comprises 10 subscales, each with a 5-point Likert score of 1 (none), 2 (mild), 3 (moderate), 4 (heavy), and 5 (serious). In totaling up the scores of each subscale and scale on mental health problems in middle-schoolers, the following was indicated: 2 to 2.99 points report a mild level, 3 to 3.99 points report a moderate level, and 4 to 4.99 points report a heavy level, and finally, a score of 5 reports a very serious level. In this study, the coefficients of subscales and overall internal consistency were 0.557 to 0.957.

## 3. Data Analysis

The study used a one-way analysis of variance (ANOVA), a descriptive analysis, and an independent sample *t*-test to examine and report the fundamental social demographic factors. Every variable had Pearson’s coefficient computation carried out. Hayes’ [54] SPSS Process Macro Model 6 was employed to obtain quantitative evidence on the serial multiple mediation effects of core self-evaluation and school adaptation in the relationship between leisure activity and cognitive function. A statistically significant *p*-value of 0.05 was used. The bootstrap confidence interval (CI) was set at 95%, and there were 5000 bootstrap samples. According to Hu and Bentler [55], the exclusion of zero in the 95% CI interval indicates a significant mediating effect.

## 4. Results

### 4.1. Common Method Bias Test

To control for common method bias (CMB), we used Harman’s [56] single-factor test in confirmatory factor analysis. Factor analysis of all the measurement indexes in the four scales of social identity, core self-evaluation, school adaptation, and mental health, and the results of unrotated factor analysis showed that there were 22 common factors with eigenvalues greater than 1, the initial eigenvalue of the first factor was 28.486, and the explained variation accounted for 26.376%, less than 40%, indicating that the deviation of the common method was not significant. Therefore, the common method deviation of the research data in this study does not exist obviously.

### 4.2. Sample Characteristics

An independent sample *t*-test and one-way ANOVA were used to evaluate the demographic factors under consideration. Table 1 displays the findings from the analysis of the study population and descriptive data. The findings demonstrated that the female cohort’s social identity was noticeably greater than that of their male counterparts (*p* = 0.000). There are significant differences in the level of social identity among migration junior high school students with different living times (*p* = 0.049). The least significant difference (LSD) was found that the social identity level of migration junior high school students with a migration time of half a year to one year was lower than that of migration junior high school students with a migration time of 3 to 5 years and more than 5 years.

### 4.3. Correlation Analysis

Social identity was significantly and negatively correlated with mental health problems (r = −0.416, *p* < 0.01). Core self-evaluation was associated with mental health issues in a negative way (r = −0.453, *p* < 0.01). School adaptation was negatively correlated with mental health problems (r= −0.635, *p* < 0.05) (Table 2).

### 4.4. Testing Core Self-Evaluation and School Adaptation as a Chain Mediator

Mediated models of core self-evaluation and school adaptation were evaluated using the SPSS 26 Process Macro Model 6 after controlling for items of gender and migration time (Table 3). Social identity had a significant positive effect on core self-evaluation (β = 0.280, t = 12.418, *p* < 0.001). Social identity (β = 0.306, t = 14.007, *p* < 0.001) and core self-evaluation (β = 0.427, t = 13.764, *p* < 0.001) were significant in positively predicting school adaptation. Social identity (β = −0.051, t = −2.133, *p* < 0.05), core self-evaluation (β = −0.154, t = −4.531, *p* < 0.001), and school adaptation (β = −0.506, t = −14.690, *p* < 0.001) had a significant negative predictive effect on mental health problems.

**Figure 1:**
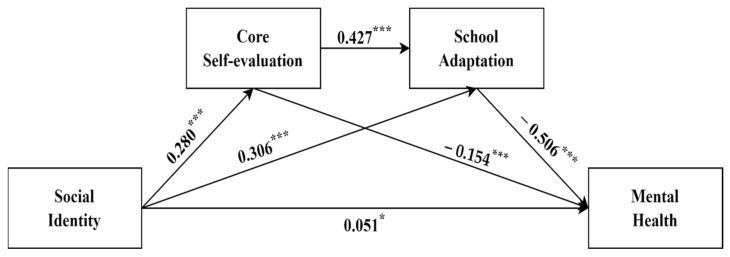
Diagram for the study variables association. *** *p* < 0.001. * *p* < 0.05.

With a bootstrapping sample of 5000, we used Hayes’ [57] SPSS 26 Process Macro Model 6 to test our hypotheses. Table 4 demonstrates that social identity negatively correlated with mental health problems (B = −0.310, *p* < 0.01). Hence, H1 was supported. In addition, after including school adaptation, the direct relationship between social identity and mental health problems was still significant (B = −0.051, *p* < 0.01), which indicated that school adaption played a partial mediating role between social identity and mental health problems (B = −0.155, *p* < 0.01). This supported H2. Furthermore, the results revealed that core self-evaluation partially mediated the negative relationship between social identity and mental health problems (B = −0.043, *p* < 0.01). This supported H3. Notably, it can also be found that when school adaptation and core self-evaluation were entered into the model, the bootstrapping analysis found that social identity had a negative impact on mental health problems through school adaptation and core self-evaluation sequentially, and its chain mediating effect was −0.061, with a 95% confidence interval not containing zero [−0.079, −0.044]. Therefore, H4 received full support (Figure 1).

## 5. Discussion and Implications

This study focused on rural-to-urban migrant school-going children in China. We sought to provide new evidence on the underpinning processes and conceptualize mechanisms that can explain the negative link between social identity and migrant children’s mental health problems. In this study, we conceptualize and operationalize a chain mediation model that presents the connections of social identity, mental health problems, school adaptation, and core self-evaluation, with the latter two variables as sequential mediators. The empirical evidence strongly supported our hypothesized chain mediation model, which highlights how social identity might affect migrant children’s mental health problems by facilitating a successful school adjustment and subsequently reinforcing their core self-evaluation. This research adds significantly to the field’s body of knowledge and has significant ramifications for educational practices, particularly in China, where an estimated 292 million rural migrant workers make up more than one-third of the country’s labor force [58]. Their children can be kept apart from their parents for years at a time and have restricted access to healthcare and education.

In addition, this study contributes to current known evidence on the consequence of migrant children’s sense of belonging to multiple groups in pre-migration; they still maintained a sense of belonging to a number of these said groups during post-migration. However, this continuity of social identities negatively impacted their mental health. Our findings are consistent with past studies that reported a negative or the absence of a direct link between belonging to multiple groups and depression [59,60]. One possible reason is that when these Chinese migrant children reminisced about their groups of friends and relatives they had left behind, it leaves a painful reminder and longing which resulted in an unhealthy mental state. Subsequently, being a member of multiple social groups pre-migration not only predicts membership continuity but also a sense of loss when uprooted from their village. This presents itself as a double-edged sword; one on hand, these children increasingly have the chance to maintain a sense of continuity, but on the other hand, they stand a chance of losing important social ties. Therefore, the consultants and social workers who serve this population need to consider which social groups occupy their thoughts the most when asked about group belonging. Different group memberships imply different meanings that could fulfill different psychological needs, thus underscoring the fact that examining the content of groups left behind is crucial [61].

Additionally, the findings of this study established the hypothesis validity of school adaptation that played a mediating role between social identity and mental health problems among Chinese migrant middle-schoolers. However, no research has been conducted on the inherent mechanism of school adaptation in migrant children’s mental health problems. The present findings have vital implications for the development of interventions and promotions that address the mental health of this concerned population. The support of key stakeholders, namely parents, teachers, and other educators, is important in improving the integration of these children’s self-identity in the destination city, thereafter halting identity conflict. Migrant children’s level of school adaption can be effectively increased by social support [62], which will improve the impact of social identity on mental health issues.

Further, our study found another important mediating path: social identity was negatively correlated with migrant students’ mental health problems through core self-evaluation. This effectively supported Dozois and Beck’s [63] cognitive model of depression (CMD), alongside some past studies that found core self-evaluation performing as a mediator between shyness and emotional disorders [64,65,66]. Those with negative core self-evaluations tended to report experiencing mental health symptoms [67]. Other studies have advocated that higher core self-evaluated individuals tend to report positive instead of negative emotions, including anxiety and depression [68]. These individuals are highly confident, actively pursuing goals that are aligned with personal values [69], and adapting well to social change and development [70]. Our results suggest that when the study cohort’s core self-evaluation is improved, their exposure and vulnerability to mental health problems can be reduced. Hence, quality core self-evaluation correctly guides Chinese migrant children to better understand their sense of ability and worth in their personal development and growth. Therefore, by exploring core self-evaluation as an influencing factor of this population, our study provides guidance and reference for key social support providers in helping these children to adapt to their school life, develop their social identities, and promote their mental health development.

## 6. Limitations and Future Research Directions

The study has some limitations that need to be acknowledged. To begin with, despite the chain mediation model in this study’s assertion that social identity, school adaptation, core self-evaluation, and mental health problems are causally related, we have yet to establish causation due to the use of a cross-sectional research design. Hence, longitudinal designs, such as cross-lagged designs or even quasi-experimental designs, should be used in future research. These might effectively scrutinize and establish concrete causality evidence of the predicted relationships. Second, to control for the common method bias (CMB) issue, attempts were made to adopt certain procedural methods, such as item clarity and anonymous assurance. However, data were gathered at the one-time point for each variable from one source, namely the Chinese migrant middle-schoolers. Thus, to address the CMB issue, future researchers should consider data collection from multiple sources. Additionally, it would serve well to separate the predictive measurement of social identity and mental health problems, and then establish criterion variables at different time points [64]. Next, our focus was limited to the chain mediating mechanism relating to social identity and mental health problems through core self-evaluation and school adaptation, but contingency factors related to this relationship were not studied. Potential boundary conditions, such as individual variations (such as extraversion and contextual variables), might be investigated to address this gap. Finally, our study sample was limited to a Chinese context despite testing a robust model of hypothesis, thus posing finding generalizability issues when operationalized in a single cultural context. Future studies could benefit from testing the model in other cultures to ascertain the applicability of our results.

## Figures and Tables

**Table 1:** Demographic characteristics of the study population and their relationship to social identity and mental health (n = 827).

Variable		N (%)	SI	F/t	MH	F/t
			(M ± SD)		(M ± SD)	
Gender	Male	465 (56.2)	4.66 ± 0.94	−3.957 ***	2.18 ± 0.73	1.846
	Female	362 (43.8)	4.92 ± 0.91		2.09 ± 0.65	
Grade	Junior One	264 (31.9)	4.74 ± 0.98	0.338	2.16 ± 0.72	2.792
	Junior Two	283 (34.2)	4.81 ± 0.87		2.06 ± 0.65	
	Junior Three	280 (33.9)	4.77 ± 0.96		2.20 ± 0.71	
Migration time (year)	0.5–1	88 (10.6)	4.58 ± 1.01	0.049 *	2.14 ± 0.69	0.038
	1–3	207 (25.0)	4.69 ± 0.96		2.13 ± 0.71	
	3–5	159 (19.2)	4.85 ± 0.89		2.15 ± 0.67	
	5>	373 (45.1)	4.84 ± 0.93		2.14 ± 0.70	

Note. Social identity = SI, Mental health = MH. *** *p* < 0.001. * *p* < 0.05.

**Table 2:** Descriptive statistics and cross-variable correlation.

No.	Construct	1	2	3	4
1	SI	1			
2	CSE	0.389 **	1		
3	SA	0.566 **	0.531 **	1	
4	MH	−0.416 **	−0.453 **	−0.635 **	1

Note. Social identity = SI, Core self-evaluation = CSE, School adaptation = SA, Mental health = MH, ** *p* < 0.01.

**Table 3:** Regression analysis of the association between study variables.

	Model 1 Outcome Variable CSE			Model 2 Outcome Variable SA			Model 3 Outcome Variable MH		
	*B*	SE	*t*	*B*	SE	*t*	*B*	SE	*t*
Gender	−0.130	0.043	−3.056 **	0.235	0.038	6.158 ***	0.064	0.039	1.669
Migration time	0.004	0.020	0.191	−0.190	0.018	−1.080	0.019	0.017	1.090
SI	0.280	0.023	12.418 ***	0.306	0.022	14.007 ***	−0.051	0.024	−2.133 *
CSE				0.427	0.031	13.764 ***	−0.154	0.034	−4.531 ***
SA							−0.506	0.035	−14.690 ***
R^2^	0.161			0.460			0.427		
*F*	52.671 ***			175.219 ***			122.373 ***		

Note. Social identity = SI, Core self-evaluation = CSE, School adaptation = SA, Mental health = MH. * *p* < 0.05, ** *p* < 0.01, *** *p* < 0.001.

**Table 4:** Mediating effect analysis of the chain mediating model.

Items	Effect Size	Boot SE	Boot CI		The Proportion of Effect Size
			**Lower**	**Upper**	
Total effects	−0.310	0.024	−0.357	−0.264	100%
Direct effects	−0.051	0.024	−0.099	−0.004	16.451%
Total indirect effects	−0.259	0.021	−0.301	−0.219	83.548%
SI → CSE→ MH	−0.043	0.011	−0.066	−0.022	13.871%
SI → SA → MH	−0.155	0.018	−0.191	−0.121	50.000%
SI → C → SA → MH	−0.061	0.009	−0.079	−0.044	19.677%

Note. Social identity = SI, Core self-evaluation = CSE, School adaptation = SA, Mental health = MH.

## Data Availability

The raw data supporting the conclusion of this article will be made available by the authors, without undue reservation.
